# Attempts to lose weight among overweight and non-overweight adolescents: a cross-national survey

**DOI:** 10.1186/1479-5868-4-50

**Published:** 2007-10-14

**Authors:** Kristiina Ojala, Carine Vereecken, Raili Välimaa, Candace Currie, Jari Villberg, Jorma Tynjälä, Lasse Kannas

**Affiliations:** 1 University of Jyväskylä, Department of Health Sciences, Research Center for Health Promotion, P.O. Box 35 (L), FIN-40014 University of Jyväskylä, Finland; 2 Department of Public Health, Ghent University, University Hospital, Bloc A, 2^nd ^Floor, De Pintelaan 185, B-9000 Ghent, Belgium; 3 Child and Adolescent Health Research Unit (CAHRU), Department of P E, Sport and Leisure Studies, University of Edinburgh, St. Leonard's Land, Holyrood Road, Edinburgh EH8 8 AQ, UK

## Abstract

**Background:**

Despite the global obesity epidemic, few studies have performed cross-national comparisons of adolescents' attempts to lose weight and weight control practices. This study aims to investigate matters mentioned above by weight status in Europe, Israel, and North America.

**Methods:**

Nationally representative samples of adolescents from over 30 countries completed an anonymous, standardized questionnaire as part of the Health Behaviour in School-aged Children 2001/2002 survey. The prevalence and likelihood of attempts to lose weight were determined. The effect of weight status, self-perception of overweight, age and country of residence upon the likelihood of current attempts to lose weight were evaluated using multilevel multivariate logistic regression in separate analyses for boys and girls. The study also presented the prevalence of weight control practices of overweight and non-overweight adolescents who had controlled their weight in seven countries.

**Results:**

In general, overweight and obese adolescents were more likely to be engaged in current attempts to lose weight and had tried to control their weight during the past 12 months more often than non-overweight adolescents. Besides weight status, self-perception of overweight and age were significant individual-level factors determining current attempts to lose weight. Country of residence was a significant second-level factor but no clear geographical pattern was found. Several gender-related differences existed.

**Conclusion:**

The findings indicated that most overweight adolescents were motivated to reduce their weight. The importance of promoting a healthy body image for all adolescents was highlighted by the fact that self-perception of overweight was found to be the most important factor leading to attempts to lose weight.

## Background

Increasing rate of childhood and adolescent obesity is a global public health concern [1]. At the same time, the stigmatization of obesity by children appears to have increased [2]. Being thin is greatly valued within Western societies and a considerable number of adolescents with normal weight, especially girls, are trying to lose weight to achieve the socially endorsed ideal of a beautiful body [3-5]. As a rule, both the prevalence and frequency of weight control behaviour, among adolescents, multiplies as the body mass index increases [3,6,7].

Extensive or long-term attempts to lose weight can have serious consequences for young people's physical and psychological development. Female dieters are more susceptible to nutritional deficiency, growth retardation, menstrual irregularities and delayed sexual maturation, irritability, sleep disturbances and concentration problems [4,5]. Extreme dieting has been connected with low self-esteem and other negative psychological states, such as a strong relationship with depression, anxiety and suicidal thoughts [5,8]. In addition, a relationship between adolescents' dieting and the development of eating disorders has been reported [9-11]. Repeated attempts to lose weight may lead to a cycle of restrictive dieting, followed by overeating or binge eating which can actually promote weight gain in adolescents. In fact, adolescent dieters were shown to have gained more weight than non-dieters during a three-year follow-up study in the USA [12].

Weight control practices among adolescents comprise a mixture of acceptable and less acceptable methods. The majority of adolescents adopt what would be considered healthy eating and exercise behaviour, but at the same time, a noticeable number use weight control methods considered to be unhealthy such as fasting, using diet pills or laxatives, vomiting and smoking [13-18]. It has been stated that overweight adolescents may adopt extreme weight reduction practices because they are further from their ideal weight or have failed to lose weight by means of modest eating or exercise changes. Some studies have also evaluated which weight control methods overweight and non-overweight adolescents use most frequently [13,19-22].

The objectives of this cross-national study are threefold: a) to determine prevalence of weight control behaviours in overweight and non-overweight 13- and 15-year-old adolescents, b) to estimate the influence of weight status on the risk of weight loss attempts and weight controlling and c) to study the contribution of gender, age, body weight status, self-perception of overweight, and country of residence on adolescents' attempts to lose weight. In addition, the study compares the use of specific weight control practices in overweight and non-overweight adolescents, who have tried to control their weight, in seven countries.

## Methods

The data was obtained from the Health Behaviour in School-aged Children (HBSC) study 2001/2002, a cross-national study that was conducted with the collaboration of the World Health Organization. The overall goal of the study is to gain new insights into, and to increase understanding of health behaviours, lifestyles, and their context in young people. The study also aims to describe and understand differences in adolescent health behaviour between various countries. Cross-sectional surveys of 11-, 13- and 15-year-old schoolchildren are undertaken every four years. The present study concentrates on adolescents. Descriptive data for the number of 13- and 15-year-old respondents are summarized in Table 1. The mean age of the respondents was 14.5 years (standard deviation 1.0) and it ranged from 13.7 in Austria to 15.1 years in Ukraine. The youngest age group of the 2001/2002 HBSC study, 11-year-olds, were excluded from the present study because questions concerning weight controlling were not presented to them. The 2001/2002 HBSC survey included a total of 36 countries or regions. All the countries carried out the data collection in accordance with the international study protocol, providing a strong basis for international comparisons [23]. Slovakia and Greenland were excluded from the present analyses because of a small sample size and Hungary because of a slightly dissimilar questionnaire when it comes to attempts to lose weight.

**Table 1:** Number of respondents, prevalence of overweight and current attempts to lose weight, missing responses on body mass index and trying to lose weight by country and gender.

	N		Overweight^1 ^%		Currently trying to lose weight %		Missings on BMI %		Missings on trying to lose weight %	
	
	Boys	Girls	Boys	Girls	Boys	Girls	Boys	Girls	Boys	Girls
Austria	1414	1425	14.8	10.29	10.6	18.3	9.1	6.7	0.6	0.4
Belgium (Fl)	1993	2143	12.5	9.4	6.0	14.2	7.3	5.9	1.0	0.6
Belgium (Fr)	1348	1536	13.8	10.3	7.8	20.7	25.5	26.5	1.0	0.8
Canada	1237	1483	24.6	15.7	8.8	25.0	11.0	13.8	0.3	0.6
Croatia	1397	1529	15.5	7.0	7.5	22.7	5.0	3.73	0.5	0.0
Czech Rep.	1586	1735	13.6	6.8	9.3	25.8	0.6	0.2	0.1	0.1
Denmark	1428	1509	11.9	10.1	11.9	33.7	11.3	10.7	1.0	1.8
England	1804	2014	17.7	14.5	9.9	23.2	31.1	34.3	1.1	0.6
Estonia	1308	1382	10.4	5.3	4.8	12.2	5.7	2.7	0.0	0.1
Finland	1740	1719	17.3	10.6	4.5	13.9	3.5	3.0	1.1	0.5
France	2722	2792	13.7	9.9	7.4	17.6	5.7	4.8	0.8	0.4
Germany	1721	1820	15.9	7.8	8.0	19.6	10.7	10.7	1.4	1.0
Greece	1243	1312	23.9	11.4	10.2	22.7	4.1	4.6	0.2	0.1
Ireland	796	1067	13.5	13.2	3.9	15.8	49.7	56.0	0.6	0.6
Israel	1584	2023	15.1	9.0	3.3	12.2	21.8	22.7	0.9	0.3
Italy	1327	1518	21.5	10.7	9.2	25.3	5.8	4.7	0.9	0.5
Latvia	1046	1224	8.8	4.1	4.9	17.2	11.7	7.9	0.8	0.5
Lithuania	1935	1842	6.4	3.8	8.4	21.8	23.1	17.8	0.1	0.2
Macedonia	1335	1442	17.1	6.9	10.6	19.9	9.3	10.3	0.8	0.6
Malta	632	708	31.3	19.8	4.5	10.4	39.3	45.3	0.3	0.6
Netherlands	1420	1372	9.5	6.6	7.8	24.4	10.1	8.7	0.9	0.7
Norway	1677	1681	15.9	9.4	7.5	23.6	9.0	9.4	1.8	1.5
Poland	2104	2131	10.5	5.0	3.4	11.5	5.7	6.0	0.2	0.0
Portugal	834	929	19.7	10.9	6.4	20.7	6.9	9.1	1.3	1.0
Russia	2532	2981	7.3	3.7	8.5	26.6	6.7	5.8	0.2	0.0
Scotland	1309	1346	16.2	12.1	5.6	15.0	46.7	51.9	0.1	0.1
Slovenia	1232	1223	18.5	10.6	7.2	16.4	4.6	2.5	0.3	0.2
Spain	1812	1909	23.9	11.8	3.3	17.9	20.0	15.6	1.0	0.5
Sweden	1215	1194	13.8	8.7	7.7	21.2	9.2	8.5	0.5	1.4
Switzerland	1535	1581	11.2	6.7	17.4	27.3	10.1	9.2	0.5	0.4
Ukraine	1321	1577	6.7	3.9	4.6	10.8	7.9	7.2	0.4	0.4
USA	1648	1898	31.7	20.2	11.2	26.3	9.9	9.2	0.4	0.3
Wales	1324	1212	23.5	17.8	5.1	12.2	24.3	15.5	0.4	0.2

Total	50965	55154	15.4	9.3	7.5	19.8	12.6	12.5	2.9	3.4

^1 ^Includes obese

The data was collected through standardised questionnaires administered by teachers in school classrooms. A cluster sample of classrooms within schools was used to achieve self-weighting samples for nationally representative estimates. Confidentiality was ensured as surveys were anonymous and respondents were assured that only group results would be reported. The questionnaire consisted of a number of mandatory questions for all participating countries and optional, additional questions [23]. The additional detailed questions on the duration of weight controlling and practiced weight control methods were included in Belgium (Flemish speaking), Canada (only duration), Estonia, Finland, Greece, Latvia, Poland and the USA.

To identify the adolescents who were trying to lose weight at the time of filling in the survey form, respondents were asked to indicate if they were at present on a diet or doing something else to lose weight. Possible responses were "Yes"; "No, but I should lose some weight"; "No, my weight is fine"; "No, because I need to put on weight". Proportions of missing responses varied from none to two percents (Table 1). The additional question "Have you gone on a diet, changed your eating habits or done something else to control your weight, during the last 12 months?" assessed the occurrence and duration of weight control practices. Six affirmative answer options for this question were from "Yes, for a few days" to "Yes, for 6 months or more". Those respondents who answered yes to this item where then asked to indicate which of the listed methods they used to control their weight during the previous 12 months. Listed weight control practices were: exercising; skipping meals; fasting (i.e. going without eating for 24 hours or more); eating fewer sweets; eating less fat; drinking fewer soft drinks; eating less (smaller amounts); eating more fruit and/or vegetables; drinking more water; restricting diet to one or more food groups (i.e. eat only fruit and vegetables, drink only, eat only bread and water); vomiting; using diet pills or laxatives; smoking more; dieting under the supervision of a professional. The elaboration of these items was based on the answers to open-ended questions about dieting practices in Belgium Flanders by 7072 adolescents [23]. Test-retest reliability of the duration of weight control during the past 12 months was good (Kappa 0.69, SE 0.05, 83% agreement) in Finnish 13- and 15-year-old pupils (N = 194), who completed the questionnaire twice with the interval of a fortnight. The kappa values of weight control practices, except eating less sweets and drinking less soft drinks, were higher than 0.60 indicating good agreement [24].

Information on height and weight were collected by asking "How much do you weigh without clothes?" and "How tall are you without shoes?" Self-reported weight and height were used to calculate the respondents' body mass index (kg/m^2^). Adolescents' weight status was categorised by means of age- and gender-specific BMI international cut-off points recommended for use in international comparisons [25]. In the present study, the group of overweight adolescents includes obese if not remarked otherwise. Prevalence of overweight (includes obese) by country are presented in Table 1. Adolescents who did not report their weight or/and height were excluded from the analysis because BMI could not be calculated (see Table 1).

Self-perceived weight was assessed on the basis of the adolescent's response to the following question: "Do you think your body is...? Much too thin; A bit too thin; About the right size; A bit too fat; Much too fat". Categories of too thin, about the right size, and too fat were created. The last category – too fat – was used to determine self-perceived overweight. Response rates for this item varied from 89.3 in Israel to 99.9 in several countries.

Prevalence rates of current attempts to lose weight, weight controlling during the past 12 months, and used weight control practices were examined according to weight status. Statistical analyses included frequencies, cross tabulations and Chi-squares. Fisher's exact test was selected when necessary due to limited number of cases in the analyses for weight control practices. Logistic regression analyses with odds ratios were carried out to assess the influence of weight status on the risk of current weight loss attempts and weight controlling during the past 12 months. A significant level of 0.05 was used for all statistical analyses and odds ratios were considered statistically significant if 95% confidence intervals did not include 1.0. Factors contributing to young people's current attempts to lose weight were analysed using a multilevel model, separately for boys and girls. Body weight status, self-perception of overweight, and age were included as variables, using one category as a reference group. To examine the effect of adolescents' country of residence, country was included in the model as a second-level factor. A median odds ratio (MOR), median value of the odds ratio between the country at highest risk and the country at lowest risk when randomly picking out two countries, was calculated to evaluate the random country effect. MOR is based on the considerations that a random country effect model regards the countries as randomly selected and therefore treats the effect of specific countries as outcomes on a random variable [26]. Multilevel modelling was carried out using MLwiN software [27].

## Results

### Prevalence of weight control behaviours by weight status

#### Current weight loss attempts

In both gender groups and in all countries, the frequency of weight loss attempts at the time of the survey was significantly higher in overweight adolescents than non-overweight adolescents: prevalence in overweight adolescents varied from 5 (Ukraine) to 46% (Denmark) among boys and from 23 (Portugal) to 76% (Denmark) among girls, whereas corresponding prevalence in non-overweight adolescents was 1 to 9% among boys and 9 to 28% among girls (Table 2).

**Table 2:** Prevalence of currently attempting to lose weight by weight status and odds ratios with 95% confidence intervals using non-overweight as a reference group.

	Current attempts to lose weight					
	
	Prevalence %		OR (95% CI)	Prevalence %		OR (95% CI)
	Non-overweight boys	Overweight^1 ^boys		Non-overweight girls	Overweight^1 ^girls	
Austria	7	27	5.06 (3.42–7.49)	16	39	3.44 (2.36–5.02)
Belgium (Fl)	4	23	7.64 (5.10–11.41)	12	37	4.39 (3.16–6.10)
Belgium (Fr)	4	26	7.94 (4.80–13.13)	15	47	4.94 (3.29- 7.40)
Canada	6	19	3.95 (2.58–6.05)	21	46	3.20 (2.33–4.38)
Croatia	3	15	5.33 (3.21–8.84)	11	28	3.25 (2.05–5.16)
Czech Rep	7	24	4.14 (2.84–6.04)	24	55	3.88 (2.65–5.68)
Denmark	7	46	10.94 (7.39–16.20)	28	76	8.32 (5.49–12.62)
England	8	23	3.41 (2.31–5.02)	20	47	3.63 (2.63–4.99)
Estonia	4	18	5.99 (3.45–10.42)	11	28	3.08 (1.78–5.31)
Finland	3	12	4.64 (2.89–7.44)	12	28	2.91 (2.03–4.18)
France	4	27	8.57 (6.26–11.72)	14	46	5.16 (3.95–6.75)
Germany	4	23	6.26 (4.20–9.31)	17	44	3.78 (2.59–5.52)
Greece	6	24	5.04 (3.41–7.46)	20	46	3.51 (2.45–5.04)
Ireland	4	18	4.96 (2.10–11.71)	20	39	2.57 (1.46–4.54)
Israel	8	31	4.86 (3.35–7.04)	26	60	4.12 (2.89–5.84)
Italy	4	21	6.70 (4.33–10.38)	20	42	2.89 (2.04–4.09)
Latvia	3	12	4.96 (2.27–10.82)	12	30	3.25 (1.69–6.24)
Lithuania	4	14	3.81 (2.00–7.24)	16	31	2.31 (1.30–4.10)
Macedonia	3	11	4.11 (2.36–4.77)	10	32	4.37 (2.68–7.11)
Malta	6	23	4.74 (2.45–9.16)	21	50	3.74 (2.22–6.30)
Netherlands	3	18	6.35 (3.58–11.27)	9	28	4.00 (2.37–6.74)
Norway	6	21	4.29 (2.91–6.32)	19	43	3.14 (2.20–4.50)
Poland	6	24	5.31 (3.64–7.73)	22	48	3.12 (2.09–4.66)
Portugal	1	11	9.62 (4.07–22.75)	10	23	2.83 (1.64–4.87)
Russia	3	10	3.20 (1.84–5.57)	15	39	3.71 (2.47–5.57)
Scotland	6	10	3.69 (2.10–6.49)	24	47	2.82 (1.74–4.57)
Slovenia	4	22	5.90 (3.79–9.21)	21	53	4.17 (2.86–6.09)
Spain	4	16	4.26 (2.83–6.43)	13	39	4.30 (3.09–5.98)
Sweden	3	20	8.42 (4.88–14.52)	13	30	2.82 (1.75–4.56)
Switzerland	5	30	8.12 (5.31–12.43)	19	56	5.43 (3.56–8.28)
Ukraine	3	5	1.65 (0.57–4.77)	17	53	5.48 (3.20–9.39)
USA	9	35	5.42 (4.07–7.23)	22	45	2.92 (2.28–3.74)
Wales	7	28	5.29 (3.63–7.71)	22	49	3.35 (2.40–4.68)

^1 ^Includes obese

**Figure 1:**
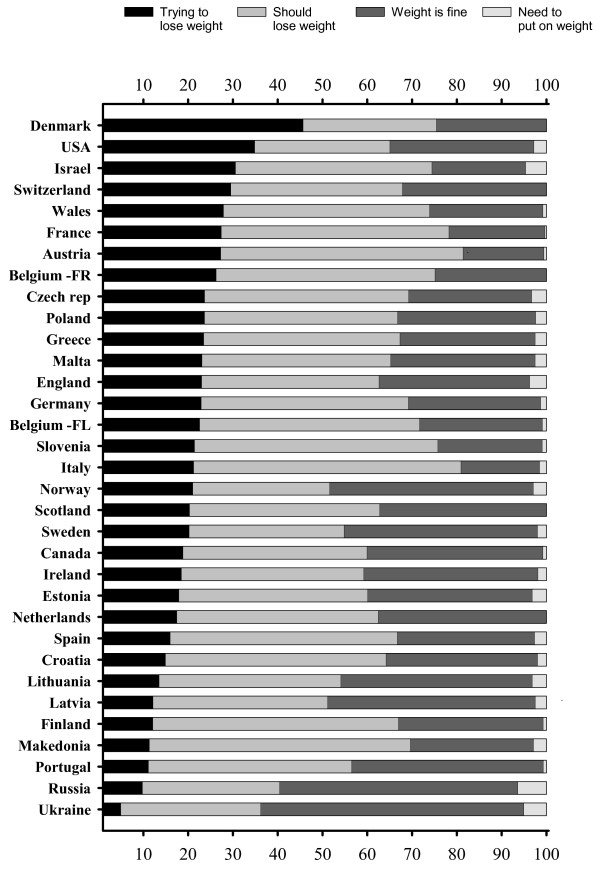
Prevalence of currently trying to lose weight, perceived need to lose or gain weight among overweight (includes obese) boys, by country.

**Figure 2:**
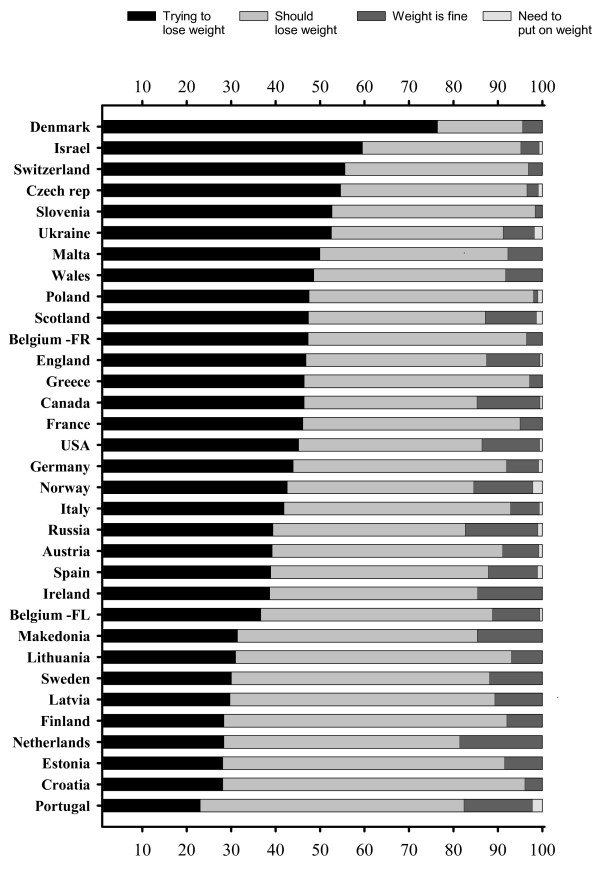
Prevalence of currently trying to lose weight, perceived need to lose or gain weight among overweight (includes obese) girls, by country.

Prevalence of trying to lose weight, perceived need to lose or gain weight and satisfaction with weight (i.e. "my weight is fine") among overweight boys and girls by country is presented in Figures 1 and 2. Overall, overweight girls tended to try to lose weight and feel the need to lose weight more commonly than overweight boys. The majority (76–98%) of overweight girls, in all countries and regions, were either currently trying or felt that they should lose some weight. By contrast, overweight boys considered their weight to be fine or wanted to gain weight (range: 19–64%) more commonly than overweight girls.

According to the international comparison, overweight boys in Ukraine, Russia, Latvia, Norway and Lithuania had the fewest attempts to lose weight, i.e. either trying to lose weight or feeling the need to lose some weight. Respectively, overweight girls in the Netherlands, Russia, Portugal, Norway, and Macedonia felt less pressure to lose weight (Figures 1, 2). It should also be noted that, in general, non-overweight adolescents who belonged to the upper 15^th ^percentile of the BMI distribution for normal weight, i.e. the heaviest adolescents with normal weight, were more generally trying to lose weight (27% vs. 11%, p < 0.001) than lighter adolescents. The result was obtained in all the countries expect Finland, Latvia, Lithuania, Norway, Portugal, Ireland, Scotland, Switzerland, Croatia, Italy, Netherlands, Slovenia, Spain, Ukraine, and Macedonia for boys and Estonia, Portugal, Ireland, Scotland, and Malta for girls.

#### Weight controlling during the previous 12 months

Overweight adolescents had gone on a diet, changed their eating habits or done something else to control their weight during the12 months leading up to the survey significantly more commonly than their normal-weight age counterparts in both genders and in all eight countries explored, with the exception of Estonian girls. The percentages of overweight girls controlling their weight exceeded that of the boys in every country; the percentages amongst the girls were over 80% in Canada, Greece, Poland and the USA (Table 3).

**Table 3:** Prevalence of weight control behaviour (i.e. do something to control weight) during the last 12 months by weight status and odds ratios with 95% confidence intervals using non-overweight as a reference group.

	Weight control behaviour					
	Prevalence %		OR (95% CI)	Prevalence %		OR (95% CI)
	Non-overweight boys	Overweight^1 ^boys		Non-overweight girls	Overweight^1 ^girls	
Belgium (Fl)	14	48	5.76 (4.28–7.74)	40	74	4.38 (3.12–6.16)
Canada	22	45	2.96 (2.21–3.96)	53	80	3.64 (2.52–5.28)
Estonia	15	34	2.83 (1.57–5.10)	49	63	1.77 (0.83–3.78)
Finland	10	31	3.89 (2.85–5.30)	45	72	3.06 (2.16–4.32)
Greece	34	61	3.05 (2.31–4.03)	60	84	3.70 (2.31–5.92)
Latvia	22	39	2.18 (1.35–3.51)	45	68	2.58 (1.38–4.81)
Poland	19	40	2.74 (1.74–4.31)	54	80	3.47 (1.77–6.79)
USA	28	59	3.76 (2.69–5.27)	56	82	3.62 (2.36–5.54)

^1 ^Includes obese

Most of the adolescents reported short-term involvement, such as less than one week, in weight control. However, a significantly greater proportion of overweight adolescents than non-overweight adolescents reported attempts to control their weight lasting over one month during the previous 12 months (data not shown). The corresponding proportions were 46% versus 37% in boys and 48% versus 32% in girls.

#### The influence of weight status on the risk of weight loss attempts and weight controlling

Overweight boys were 3.20 (Russia) to 10.94 (Denmark) times more likely to currently try to lose weight compared to non-overweight boys. The lowest OR among boys was found in Ukraine; however, the result was not statistically significant. Corresponding ORs for girls varied from 2.31 in Lithuania to 8.32 in Denmark. In all surveyed countries except England, Macedonia, Russia, Spain and Ukraine, the overweight adolescents' ORs for current weight loss attempts were greater for boys than for girls. Denmark, France, Switzerland, and both French and Flemish speaking Belgium were in the top quartile for the highest odds ratios among both genders. Nevertheless, no clear geographical pattern was found (Table 2).

Overweight adolescents' ORs for weight controlling during the past 12 months were higher than those for non-overweight adolescents in both genders and all surveyed countries with exception of Estonian girls: overweight boys had gone on a diet, changed their eating habits or done something else to control their weight from 2.18 (Latvia) to 5.76 (Belgium Fl) and overweight girls from 2.58 (Latvia) to 4.38 (Belgium Fl) times more likely than their normal-weight age counterparts. The highest odds ratios for weight controlling during the past 12 months was found in Flemish speaking Belgium among both genders.

#### Contributing factors for attempts to lose weight

Results of a multilevel model for pooled samples for 49930 boys and 52587 girls showed that both individual factors and nationality contributed significantly to the variation in current attempts to lose weight. Self-perception of overweight was the most effective individual-level factor (boys: OR = 9.44, p < 0.001; girls OR = 5.95, p < 0.001, reference group adolescents who thought their body was about the right size). Self-perception of overweight was followed by body weight status and age. Boys, whose BMI exceeded the age specific cut-off points for overweight but not obesity, were 1.54 times (p < 0.001) and obese boys 1.89 times (p < 0.001) more likely to try to lose weight than non-overweight boys. The corresponding ORs for girls were 1.62 (p < 0.001) and 1.95 (p < 0.001).

Interestingly, the association between age and current attempts to lose weight was opposite among the genders. Boys of 13-years of age were 1.19 (p = 0.018) times more likely to try to lose weight compared to boys of 15-years of age, whereas attempts to lose weight were more prevalent in the older age group among girls (OR = 1.29, p < 0.001).

In addition to variance between adolescents, the median value of the odds ratio between the country with the highest risk and the country with the lowest risk, when randomly selecting two countries, demonstrated a clear effect of country on current attempts to lose weight. The MOR for boys was 1.51 and for girls 1.43.

#### Specific weight control practices used during the previous 12 months by weight status

Prevalences of Table 4 and 5 indicate how many percent of the non-overweight and overweight adolescents, who have tried to control their weight at one point or another during the 12 months leading up to survey, reported to have used the listed weight control practices. It should be noted that prevalences are not overall pervalences. Exercising (range 71–97%) and eating fewer sweets (28–100%) were the most commonly indicated weight control practices among both overweight and non-overweight adolescents in all countries and both genders, with the exception of Latvian overweight girls. Unhealthy practices such as fasting (range 4–30%), vomiting (1–14%), diet pills or laxative use (0–19%) and smoking more (3–17%) were less frequently, but still considerably, mentioned.

Prevalences of the weight control practices among overweight adolescents who had tried to control their weight exceeded those among non-overweight adolescents with exception of exercising in every country, vomiting in six countries out of seven and occasional weight control practices in some countries. The difference in used weight control practices between overweight and non-overweight weight controllers was significant in most of the countries with regard to eating fewer sweets, drinking fewer soft drinks and dieting under the supervision of a health care professional as a means to control weight. Statistically significant differences between non-overweight and overweight weight controllers occurred most often among Greek boys. (See additional file 1: Table 4 Prevalence of weight control practices of boys who tried to control their weight during the last 12 months by country and weight status, Table 5 Prevalence of weight control practices of girls who tried to control their weight during the last 12 months by country and weight status).

## Discussion

It is vital to support the development of appropriate obesity prevention strategies and the promotion of healthy weight control practices, especially among overweight youth. This study aimed to examine adolescents' attempts to lose weight by weight status and assess the influence of gender, age, body weight status, self-perceived overweight and country of residence on these attempts. In addition, the influence of weight status on the risk of weight loss attempts and weight controlling were examined and the distribution of selected weight control practices among overweight and non-overweight 13- and 15-year-old weight controllers was presented.

Most of the overweight adolescents seemed motivated to reduce their weight. According to the results, they attempted to lose weight, felt they needed to lose weight, had gone on a diet, changed their eating habits, or done something else to control their weight during the past 12 months more frequently than non-overweight adolescents. The results confirmed that depending on the considered country and gender, overweight adolescents were three to eleven times more likely to currently try to lose weight and two to six times more likely to have done something to control their weight during the 12 months leading up to the survey than non-overweight adolescents. Additionally, overweight adolescents reported long-term attempts to control their weight more frequently than their normal-weight age counterparts and according to the answers of specific weight control practices, were aware of several methods to control weight. It may be that the increased bias against obesity drives obese young people to turn to rapid or unhealthy ways to lose weight [2]. It is therefore essential to promote self-esteem for all health-related behaviours and particularly, for weight control. Young people who value their body and health are less likely to engage in rapid or extreme weight reduction practices, regardless of their body weight [3].

Gender-related differences were evident in the present study. The results suggested that gender differences were rather parallel cross-culturally. Compared to boys, both non-overweight and overweight girls were more commonly trying to lose weight and had controlled their weight during the 12 months leading up to the survey. Overweight girls were acutely aware of their overweight in all the countries surveyed – at least 80% of them were either trying to lose weight or felt that they should lose weight. Variation among boys was greater. A small proportion of overweight boys even reported that they want to put on weight. The result may be a question of a slight misinterpretation. For boys, "put on weight" can mean to gain muscles. Males are most likely to report dissatisfaction with their muscle size and shape whereas females are more often dissatisfied with their weight and want to become thinner, even regardless of their weight [3,7,28-31]. Self-perception of overweight due to the more intense cultural pressure to be thin among females partly explains relatively high percentages of non-overweight girls trying to lose weight. It is also worth remembering that the 13- and 15-year-old girls may see the increase in weight caused by physical development as an obstacle for reaching the ideal thin female body [31]. This might be an explanation to why current attempts to lose weight were more prevalent in the older age group among girls but in younger age group among boys. In addition, smaller median odds ratio for girls' than boys' current attempts to lose weight imply that the cultural influences of slimness are more coherent for females than males. As an example from the present study, most of the Ukrainian overweight boys felt that their weight was fine or wanted to put on weight but overweight girls' opinions of their weight did not substantially differ from the rest of the European countries. The above assumptions support Bilunka's and Utermohlen's [32] finding that the spread of the Western thin ideal body has reached Ukrainian females.

McElhone and her co-authors [33] studied the cultural differences regarding the ideal thin body within a representative sample in the European Union. They reported the percentages of adult subjects selecting underweight body image figures as their ideal body weight as well as the percentages of subjects selecting normal body images as their ideal. They expressed these percentages as ratios and they found them to be highest in Greece, Italy and France. Interestingly, in the present study adolescent weight control behaviour during the past 12 months leading up to the survey was most common in Greece. Furthermore, Italian and French overweight boys were particularly aware of their overweight: about 80% of them was either currently trying to lose weight or felt that they need to lose weight.

Adolescents' country of residence had a significant effect on their attempts to lose weight according to the multilevel modelling despite the lack of an existing clear geographical pattern. The actual cause of this variation can only be speculated. It has been stated that national campaigns held with the aim of reducing weight through increased exercise and a low-fat diet may reflect a higher level of dissatisfaction and, by extension, weight loss practices in these countries [28,33]. The highest likelihoods for the current attempts to lose weight in overweight adolescents compared to non-overweight adolescents were found in Denmark, France, Switzerland, and Belgium. Unfortunately, at present there is no data available on possible campaigns running in the European countries.

The presented results should obviously be seen in light of the weaknesses and strengths of the study. The primary limitation of this study was that body weights and heights were derived from self-reports. Although this raises questions about the accuracy of the BMI values, previous studies have shown that the rates of overweight derived through self-reporting measures are fairly reliable and classification as normal and overweight can be done quite accurately [34,35]. However, Tsigilis [36] and Elgar et al. [37] stated that adolescents' self-reported and measured height and weight are highly correlated but a bias of underreporting of body weight will contribute to an underestimation of the prevalence of overweight. Therefore the prevalence of overweight may be higher than reported herein. A second limitation was a great number of missing weight or height data in some countries, namely England, Ireland, Malta and Scotland. However, Janssen et al. [38] found that weight loss practices were similar both for those who reported their height and weight and for those who did not. In comparisons by weight status, the low prevalence of overweight together with limited sample sizes can be considered as a third limitation. A fourth limitation was that adolescents may interpret the concepts of being on a diet and weight controlling differently than do adults or health professionals. In many cases adolescent girls perceive dieting and healthy eating in a similar way [39,40]. Unfortunately the design of the present survey did not, for example, allow for interviews about each respondent's conceptions.

Despite some weaknesses in the present study, the benefits of a wide international cross-sectional study on adolescents' attempts to lose weight and weight control practices should not be underestimated. The major strength of this study is the comparable data on adolescents from over 30 countries. Our results indicate, for instance, the importance of self-perceived overweight and conspicuous gender differences. However, some of the international findings require further investigation. For example, what are the specific factors within countries that bring about country-level contribution in current attempts to lose weight? Additionally, in order to promote healthy practices in controlling weight the adolescents would need information that is targeted and presented in a way which appeals to them. This means a study that is both structured and communicated with methods and means that adolescents can relate to. The holistic and critical examination of life-style choices together with the youth may give them valuable tools for everyday decisions.

## Conclusion

The international data demonstrated that adolescents' attempts to lose weight are not only strongly defined by weight status but also by gender, age, self-perception of overweight and the country of residence. The development of a joint European and North American action to raise adolescents' awareness regarding the benefits of healthy weight control practices is especially recommended because the key elements – lifelong adequate physical activity and nutrition – are globally similar.

## Abbreviations

BMI: Body mass index

Fl: Flemish speaking

Fr: French speaking

HBSC: Health Behaviour in School-aged Children study

OR: Odds ratio

MOR: Median odds ratio

95% CI: 95% confidence interval

SE: Standard error

## Competing interests

The author(s) declare that they have no competing interests.

The questions that were asked of all authors:

• In the past five years, have you received reimbursements, fees, funding, or salary from an organization that may in any way gain or lose financially from the publication of this manuscript, either now or in the future? No.

• Do you hold any stocks or shares in an organization that may in any way gain or lose financially from the publication of this manuscript, either now or in the future? No.

• Do you hold or are you currently applying for any patents relating to the context of the manuscript? Have you received reimbursements, fees, funding, or salary from an organization that holds or had applied for patents relating to the content of the manuscript? No.

• Do you have any other financial competing interests? No.

## Authors' contributions

KO drafted the manuscript, developed its design further, and performed some of the statistical analyses.

CV conceived of the study questions and helped to draft the manuscript.

RV and CC edited the manuscript.

JV participated in the design of the study and performed most of the statistical analyses.

JT and LK revised the manuscript critically.

All authors have read and approved the final manuscript.

## Supplementary Material

Additional file 1 **Additional tables on weight control practices**. Additional file includes following tables: Table 4 Prevalence of weight control practices among overweight and non-overweight boys who tried to control their weight during the last 12 months by country, and Table 5 Prevalence of weight control practices among overweight and non-overweight girls who tried to control their weight during the last 12 months by country. Click here for file
