# Supplementary material for: Attempts to lose weight among overweight and non-overweight adolescents: a cross-national survey
Source: Int J Behav Nutr Phys Act. 2007 Oct 14;4:50. doi: 10.1186/1479-5868-4-50 (PMC2174511; doi:10.1186/1479-5868-4-50)
Supplement: Additional file 1 — Additional tables on weight control practices. Additional file includes following tables: Table 4 Prevalence of weight control practices among overweight and non-overweight boys who tried to control their weight during the last 12 months by country, and Table 5 Prevalence of weight control practices among overweight and non-overweight girls who tried to control their weight during the last 12 months by country. [file 1479-5868-4-50-S1.pdf]

Additional file 1: Additional tables on weight control practices

Table 4 Prevalence of weight control practices among non-overweight and overweight boys who tried to control their weight during the last 12 months by country.

	%													
	Belgium (Fl)		Estonia		Finland		Greece		Latvia		Poland		USA	
(n)	N[1] (223)	O[2] (110)	N (81)	O (20)	N (137)	O (86)	N (301)	O (170)	N (185)	O (31)	N (168)	O (36)	N (118)	O (137)
Exercise	84	78	86	80	90	81	85	88	78	77	93	92	97	95
Less sweets	80	90*	28	45	73	88**	67	78*	60	83*	50	77**	73	79
More water	73	82	38	30	56	75**	64	76**	57	73	55	43	80	79
More fruit and/or vegetables	64	71	46	35	67	71	67	66	68	90**	62	51	66	54
Less fat	59	76**	30	55*	67	84**	64	71	68	80	45	66*	58	71*
Less soft drinks	59	76**	28	60**	56	71*	49	67**	49	34	34	40	48	65**
Smaller amounts	44	55	26	35	40	60**	60	77**	44	76**	39	71**	46	73***
Skipping meals	21	22	18	25	31	38	46	59**	29	52**	32	60	39	45
Restrict diet	11	12	11	25	16	12	12	13	25	24	26	26	7	13
Fasting	4	5	5	5	10	6	18	11	16	14	9	6	14	17
Smoking	7	11	9	15	9	10	5	8	16	3	10	9	10	10
Diet pills, laxatives	2	6*	1	0	3	2	0	1	12	0	4	11	8	8
Vomiting	3	1	4	5	7	4	4	1	14	3	2	3	4	3
Dieting under supervision of a professional	5	13*	2	0	6	5	6	15**	11	4	2	11**	3	10*

[1] Non-overweight, [2]Overweight (includes obese)
Analysis between non-overweight and overweight adolescents was performed with a Chi-square or Fisher's exact test.
***p<0.001
**p<0.01
*p<0.05

Table 5 Prevalence of weight control practices among non-overweight and overweight girls who tried to control their weight during the last 12 months by country.

	%													
	Belgium (Fl)		Estonia		Finland		Greece		Latvia		Poland		USA	
(n)	N[1] (723)	O[2] (140)	N (300)	O (19)	N (656)	O (123)	N (647)	O (120)	N (486)	O (32)	N (534)	O (45)	N (352)	O (136)
Exercise	78	72	86	79	91	91	74	72	75	71	90	86	93	90
Less sweets	91	94	77	84	89	95*	80	90	81	96*	84	100**	86	86
More water	83	88	68	74	77	85	75	88**	69	85	64	68	84	84
More fruit and/or vegetables	72	76	67	32**	79	84	75	76	76	85	77	93**	62	70
Less fat	76	81	71	74	82	88	76	81	84	92	78	79	81	80
Less soft drinks	72	82*	57	58	74	86**	71	81*	57	84**	60	68	72	80*
Smaller amounts	66	75	66	63	66	71	72	82*	72	79	80	98***	80	84
Skipping meals	35	37	48	47	53	47	57	65	50	56	69	81	62	72**
Restrict diet	16	18	18	16	24	22	12	16	17	26	32	45	16	27**
Fasting	6	6	13	5	12	9	11	14	18	14	14	19	25	30
Smoking	11	17	12	10	12	13	5	5	8	9	11	10	11	15
Diet pills, laxatives	4	9 **	6	10	2	3	0	2	4	13	15	17	14	18
Vomiting	5	3	7	10	10	10	2	2	5	8	10	12	8	8
Dieting under supervision of a professional	6	20***	2	5	0	4*	12	27***	5	14	1	12***	5	10*

[1] Non-overweight, [2]Overweight (includes obese)

Analysis between non-overweight and overweight adolescents was performed with a Chi-square or Fisher's exact test.

***p<0.001

**p<0.01

*p<0.05
